# Sugar-inducible promoters for manipulation of core metabolic pathways in the thermophilic acetogen *Thermoanaerobacter kivui*

**DOI:** 10.1128/aem.01641-25

**Published:** 2025-11-20

**Authors:** Benjamin Zeldes, Sabina Mittelstedt, Christoph Baum, Adilia Shakirova, Anja Poehlein, Rolf Daniel, Mirko Basen

**Affiliations:** 1 Microbiology, Institute of Biological Sciences, University of Rostock 98915 , Rostock, Germany; 2 Genomic and Applied Microbiology & Göttingen Genomics Laboratory, Georg-August-University 9375, Göttingen, Germany; Kyoto University, Kyoto, Japan

**Keywords:** thermophile, acetogen, inducible promoters, Wood-Ljungdahl pathway, metabolic bottleneck

## Abstract

Acetogenic bacteria form an essential part of the global carbon cycle and show promise for green chemicals production thanks to their energy-efficient carbon fixation via the Wood-Ljungdahl pathway. Energy conservation in acetogens is dependent on one of two ion-pumping membrane complexes, Rnf or Ech. While the energetics of Rnf acetogens are relatively well understood, Ech-dependent metabolism remains unclear. Like many Ech acetogens, *Thermoanaerobacter kivui* (T_opt_ = 66°C) encodes two separate Ech complexes and is genetically tractable, allowing it to serve as a model of Ech-dependent metabolism. While the role of the Ech2 complex has been determined biochemically and by gene knockout, Ech1 has eluded biochemical characterization, and knockout attempts have been unsuccessful, suggesting it is essential. This work identifies two sugar-inducible promoters with very low expression under non-inducing conditions and uses them to replace the native Ech1 promoter. The resulting strains exhibit reduced growth in non-inducing “knockdown” conditions, confirming the importance of Ech1 for metabolism. Additionally, knockdown of the gene encoding formyl-THF synthetase (Fhs)—which consumes the metabolic intermediate formate—resulted in significantly more formate accumulating in the culture medium. Together, these results confirm that the characterized promoters can be used to elucidate the function of essential genes not amenable to knockout.

**IMPORTANCE:**

Acetogenic bacteria are industrially relevant for conversion of synthesis gas (syngas, a mixture of hydrogen, H_2_; carbon monoxide, CO; and carbon dioxide, CO_2_) to products. Thermophilic bacteria have long been of interest as industrial strains due to their resistance to contamination. *Thermoanaerobacter kivui* is among the most thermophilic acetogens (T_opt_ = 66°C), with very short doubling times on H_2_+CO_2_ (&lt; 2 h), and is therefore a prime candidate for synthesis gas conversion at high temperatures. Here, we expanded *T. kivui*’s genetic toolkit by establishing a reporter gene operating at its temperature optimum, which was used to characterize sugar-inducible promoters. The identified promoters were used to control and engineer the metabolism of *T. kivui*, and may now be applied to elucidate remaining mysteries about the energetics of acetogenic metabolism.

## INTRODUCTION

Acetogens fix CO_2_ via the Wood-Ljungdahl pathway (WLP), the most energy-efficient carbon fixation pathway (1). WLP-utilizing organisms eke out the final bits of energy left over at the end of anaerobic digestion and can serve as primary producers in energy-constrained environments, such as sediments (2) and the deep subsurface (3). Acetogens play an important role in animal nutrition (4, 5) and are of particular biotechnological interest for the conversion of waste industrial gasses to carbon chemicals (6). *Thermoanaerobacter kivui* is the most thermophilic acetogen characterized to date (T_opt_ = 66°C) and is capable of being adapted to utilize CO (7), making it a candidate for conversion of industrial syngas (containing H_2_, CO_2_, and CO). It also grows on a limited set of simple sugars, including glucose, fructose, mannose (8), and on the sugar alcohol mannitol (9).

**Fig 1:**
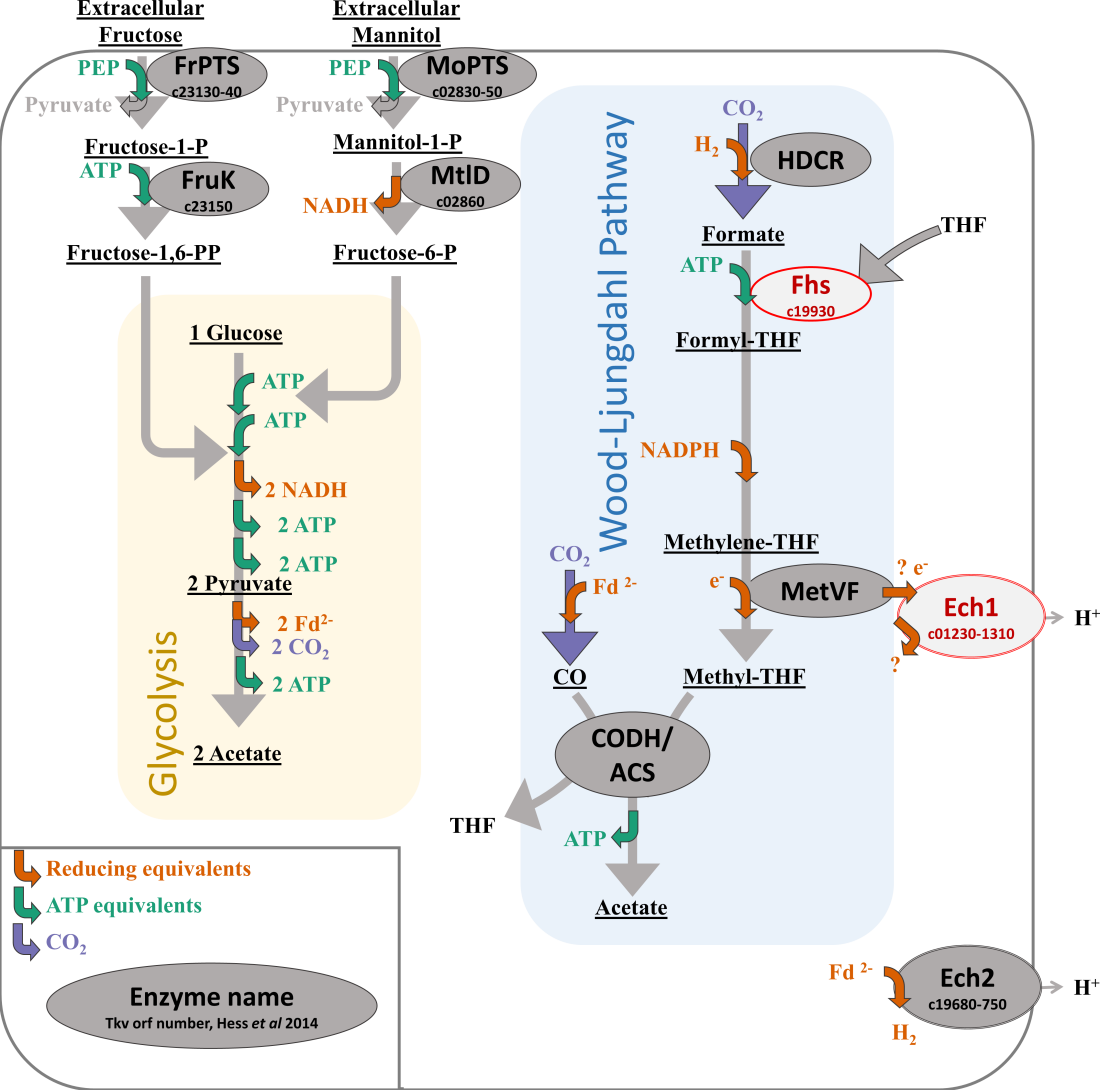
Simplified metabolic map of *T. kivui*, highlighting sugar metabolism and enzyme targets for knockdown. Selected enzymes and metabolites are indicated, and encoding gene OFR#s (Tkv_) relevant to the text are included. Colored arrows indicate production or consumption of reducing equivalents (orange), ATP equivalents (green), and CO_2_ (purple). Cases where the specific reducing equivalent is unknown are indicated with “e^–^,” and possible electron transfer between MetVF and Ech1 is indicated with “?.” Enzyme candidates for gene “knockdown” by replacing the promoter region with an inducible promoter are indicated in red.

Most genes of the WLP in *T. kivui* are clustered together in a large operon starting with formyl-tetrahydrofolate (THF) synthetase (*fhs*), which activates formate (generated by reducing CO_2_) to formyl-THF (10). Formate shows promise as a medium for green H_2_ storage (11), or as a feedstock for bioproducts (12). Knocking out one of two *fhs* genes in the mesophilic acetogen *Acetobacterium woodii* resulted in a severe growth defect and in accumulation of formate during growth on H_2_+CO_2_ (13). Knockout of *fhs* in *T. kivui* has also been proposed as a way to maximize formate production (14), but as *T. kivui* has only one copy of *fhs* its deletion is likely to be lethal. Therefore, we envisioned placing *T. kivui*’s *fhs* under the control of an inducible promoter to study growth and formate production (Fig. 1).

For energy conservation, the WLP must be coupled to a transmembrane gradient via either an Rnf or Ech transmembrane complex (15). The energetics of Rnf-containing acetogens have been elucidated, but gaps remain in our understanding of Ech-containing acetogens like *T. kivui*, which contains two *ech* gene clusters but no Rnf. The Ech2 complex has been purified and confirmed to function as a proton-translocating ferredoxin-dependent hydrogenase (16). Overexpression of the *ech2* operon has been described to improve growth of *T. kivui* on CO (17), while a ∆*ech2* knockout strain that grew well on H_2_+CO_2_ or sugars was unable to grow on CO, suggesting Ech2 is essential for ferredoxin recycling on this substrate (18). Ech1 is hypothesized to accept electrons directly from the methylene-THF reductase MetVF of the WLP (19), which would make it the key coupling point between the WLP and proton-motive-force energy conservation. The *ech1* gene cluster could not be deleted after numerous attempts, suggesting that it may be essential. We hypothesized that this essentiality could be overcome by replacing its native promoter with an inducible promoter (Fig. 1). The ideal promoter for this application would allow a strong binary comparison between very high induced expression and very low non-induced “knockdown” expression.

Several model thermophiles have promoters available that are induced by the presence of a specific sugar, such as maltose in *Sulfolobus acidocaldarius* (20), xylose in *Anaerocellum* (*f. Caldicellulosiruptor*) *bescii* (21), cellobiose in *Thermus thermophilus* (22), and various sugars in some *Geobacillus* species (23). Induction without sugar is rarer but has also been demonstrated in thermophiles, for example, by iron starvation (24). Inducible promoters are established for several mesophilic acetogens (25), but so far, the only promoters confirmed to function in thermophilic acetogens are constitutive (26, 27). Promisingly, essential genes involved in metabolism of mannitol (*mtlD*) and fructose (*fruK*) in *T. kivui* have been identified by gene knockouts (9, 28) (Fig. 1). Both genes occur in operons also containing components of phosphotransferase system (PTS) sugar-specific EII uptake genes and a regulatory gene, which likely controls expression of the entire operon in response to the presence of the respective sugar. Sugar-inducible promoters typically exhibit strong binary on/off response, with increased expression evident at inducer concentrations in the low micromolar (µM) range, and by 1 mM inducer, maximal expression is reached (29). Therefore, sugar-inducible promoters are ideal for the generation of our proposed “knockdown” strains.

To quickly characterize promoter strength, fluorescent reporter systems such as GFP are ideal, but while thermostable GFP derivatives are available, they do not function under anaerobic conditions (30). Systems that do not require molecular oxygen have been developed, including the Y-FAST protein, which binds small externally supplied fluorophores (31). This system has been successfully applied to mesophilic anaerobes, including acetogens (25). The Y-FAST system has even been applied in *T. kivui*, but required sub-optimal growth temperatures and resulted in reduced sensitivity that prevented characterization of all but the strongest promoters (27). In contrast, β-galactosidase is native to several extreme thermophiles, and has been used as a reporter gene for promoter characterization at or above 70°C (20, 32).

The goal of this study was to expand the repertoire of promoters available to control gene expression in *T. kivui*. A global transcriptional analysis on glucose and mannitol was carried out with RNA-sequencing, and expression of the mannitol and fructose gene operons during growth on various sugars was monitored by quantitative RT-PCR. The respective promoter regions from these operons were tested, first for their ability to control expression of a recombinant β-galactosidase reporter gene from *A. bescii*, and then to control native genes believed to be essential for acetogenic metabolism (*fhs* and the *ech1* operon). The utilization of the inducible promoters described here represents a proof of concept towards their application in gas “fermentation” with *T. kivui*.

## RESULTS

### RNA sequencing of glucose and mannitol grown cells

Induction of mannitol metabolism by mannitol has been reported in *T. kivui* before (9). Therefore, we decided to carry out a differential RNA analysis to obtain information on putative sugar-inducible promoters and underlying regulatory mechanisms.

Wild-type *T. kivui* cells were grown to late exponential phase in complex medium containing either 25 mM glucose or 25 mM mannitol, and cells harvested for RNA sequencing. Principal component analysis showed good separation of the two sugar conditions (Fig. S1). Changes in gene expression were considered statistically significant if the adjusted P_value_ (Padj) was less than 0.001, resulting in a total of 19 genes upregulated on mannitol relative to growth on glucose (log2 fold-change >2) and 14 genes upregulated on glucose relative to mannitol (log2 fold-change < −2) (Table S1 and Table S2).

The mannitol gene operon identified previously (TKV_c02830-60) was upregulated on mannitol roughly fivefold (log2-fold = 2.2 to 2.6). Other genes upregulated on mannitol and likely involved in sugar metabolism included *pfp* (Tkv_c18810), encoding a pyrophosphate-specific alternative to the ATP-dependent phosphofructokinase, and a transcriptional regulator annotated as xylose repressor *xylR* (Tkv_c10940). In addition, gluconeogenic enzymes phosphoenolpyruvate synthase *ppsA* (Tkv_c10530) and fructose 1,6-bisphosphatase (Tkv_c02390) were upregulated on mannitol. The most strongly upregulated genes on mannitol were ferrous iron transport proteins A and B (TKV_c01950-60).

Genes upregulated on glucose included a cluster of tryptophan biosynthesis genes (Tkv_c14840-90), and parts of a gene cluster annotated as involved in iron-molybdenum cofactor biosynthesis (Tkv_c17530-50). *T. kivui* grows faster on glucose than on mannitol, and the upregulation of amino acid and cofactor biosynthesis may reflect greater need for these components due to the higher growth rate. Desulfoferrodoxin (Tkv_c22020) plays a role in detoxification of radical oxygen species (33) generated at higher rates during more rapid growth.

While not differentially regulated, the gene for the S-layer protein (*slp*) (TKV_c23170) is the single most strongly expressed transcript under both conditions, confirming the status of P_slp_ as a very strong constitutive promoter. Transcript levels for the phosphate acetyltransferase *pts* (TKV_c13970), recently described to have an even stronger constitutive promoter (27), were an order of magnitude lower (Table S2). Key genes of acetogenic metabolism were not differentially regulated but had high baseMean levels, including *fhs* (TKV_c19930), the first gene of the WLP operon, and *ech1A* (TKV_c01230). The genes of the Ech2 operon, starting with *ech2D* (TKV_c19750), were expressed at substantially lower levels than Ech1, as reported previously (18).

### Growth of *T. kivui* on mixed sugars

**Fig 2:**
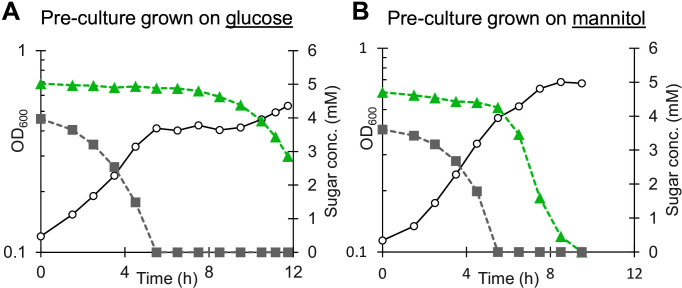
Consumption of mannitol is repressed by glucose in *T. kivui*. Cells pre-cultured on (**A**) glucose or (**B**) mannitol were passaged onto complex medium at 66°C containing both glucose and mannitol (5 mM each). Optical density is shown in open circles, glucose concentration in gray squares, and mannitol concentration in green triangles. One representative sample of biological duplicates is shown.

*T. kivui* grows well on glucose and fructose (8). Growth on mannitol is slower and suffers from a very long lag phase unless the pre-culture was also grown on mannitol (9), suggesting mannitol is a less-favored growth substrate. To investigate this further, wild-type *T. kivui* cells were pre-cultured on either mannitol or glucose, then transferred into complex media with a mixture of both glucose and mannitol (Fig. 2). Cells rapidly consumed the glucose in the media, but cells pre-cultured on glucose exhibited very strong diauxic growth: upon depletion of glucose, growth stopped completely and did not resume until several hours later when mannitol consumption finally began (Fig. 2A). This may be explained by the presence of a carbon catabolite repression system in *T. kivui*, or by a longer adaptation to mannitol (9). In cells pre-grown on mannitol, cells started rapidly consuming mannitol as soon as glucose was depleted (Fig. 2B).

### Expression of mannitol and fructose genes on mixed sugars

**Fig 3:**
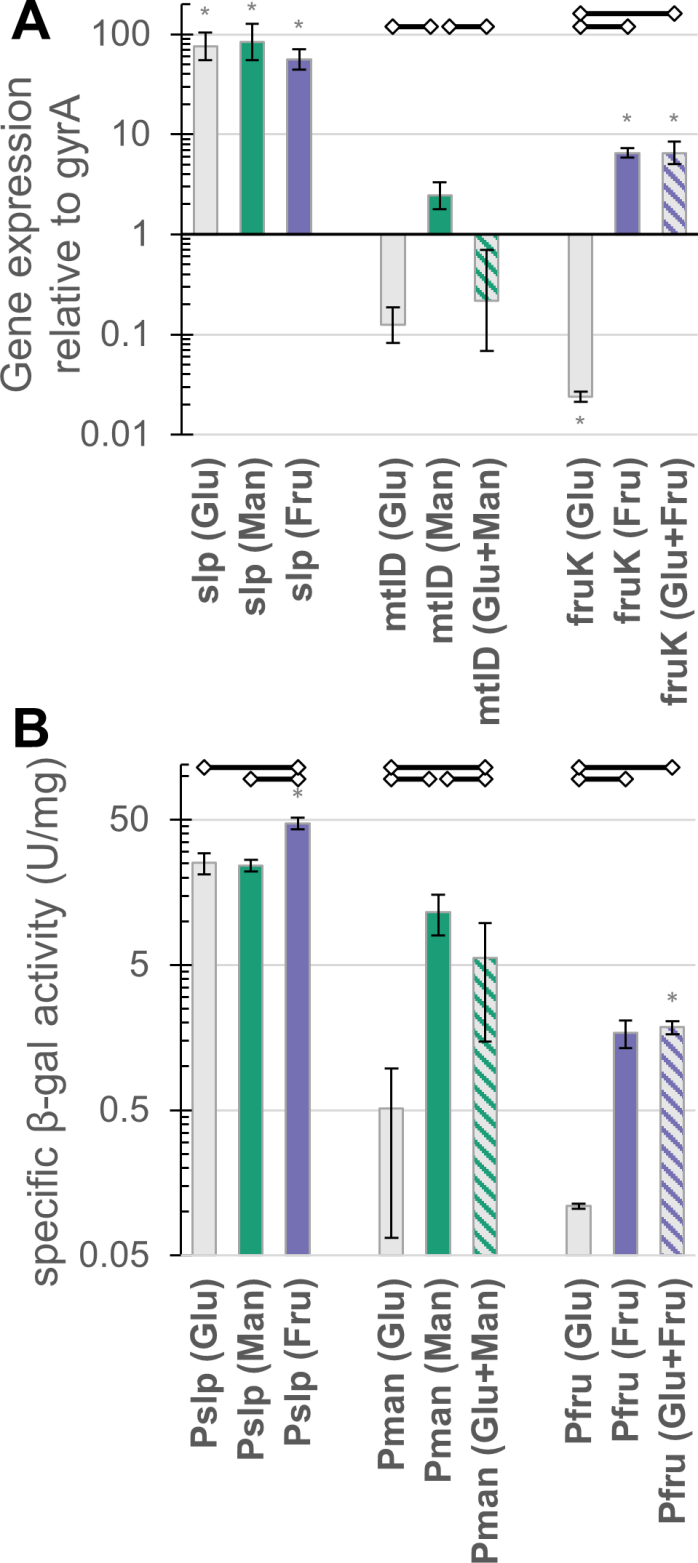
Genes of the mannitol and fructose operons are up-regulated in response to the respective sugar. (**A**) Expression level of *slp*, *mtlD,* and *fruK* genes in wild-type *T. kivui* relative to expression of the *gyrA* gene [2^(-∆Ct)]. (**B**) β-galactosidase activity in cell extracts of mutant strains of *T. kivui* expressing a thermostable β-galactosidase under the control of the three promoters being tested. Cells were grown in defined or complex medium on various sugars (30 mM) as indicated by bar color (gray = glucose, green = mannitol, purple = fructose, while hashed bars are mixtures of two sugars). Cells were grown at 66°C and β-galactosidase activity assayed at 65°C. Each bar represents the average of biological replicates, error bars indicate standard deviation, except for bars marked with “*” indicating only two replicates, where error bars represent minimum and maximum measured values. Open diamonds connected by black lines indicate comparisons that are significantly different (*P* < 0.05).

Since the genes *mtlD* (Tkv_c02860) and *fruK* (Tkv_c23150) are essential for growth on mannitol and fructose, respectively, expression levels of these two genes and *slp* were measured on various sugars using qPCR (Fig. 3A). As expected, expression of *slp* was extremely high under all growth conditions, while *mtlD* was upregulated during growth on mannitol. Maximal expression of *mtlD* was on mannitol, at 2.5-fold higher than the reference *gyrA* gene, while minimal expression was on glucose at eightfold below the reference gene, implying a roughly 20-fold upregulation on mannitol, vs between four- and sevenfold based on RNAseq (Table S2). In line with the catabolite repression hypothesis, the addition of glucose eliminated the mannitol-induced upregulation.

*fruK* exhibited even higher induced expression (6-fold above *gyrA*) on fructose, and even lower basal expression (40-fold lower than *gyrA*), for an upregulation on fructose more than 250-fold relative to growth on glucose (Fig. 3A). Upregulation of *fruK* is also evident in cultures grown on a mixture of glucose and fructose, indicating fructose utilization is not repressed by the presence of glucose. Additional tests with all combinations of sugars used by *T. kivui* (glucose, fructose, mannitol, and mannose) indicated that *mtlD* was repressed by all other sugars, while *fruK* expression was unaffected (Fig. S2). Since the fructose operon exhibited lower leakiness and higher induced expression than the mannitol operon and was induced even in the presence of other sugars, the fructose promoter seemed to be the best candidate for a sugar-inducible promoter, while the mannitol promoter was of interest due to its ability to be repressed by simple addition of another sugar substrate.

To confirm that the observed transcriptional responses in the native genes translate to heterologous protein expression, the β-galactosidase (β-gal) of the extremely thermophilic, plant biomass deconstructing *A. bescii* (34, 35) was cloned into the *T. kivui* genome as a reporter gene, under the control of the three promoters being tested (referred to as strains P_slp_, P,_fru_ and P_man_ based on which promoter they contain). The promoter strains were cultured on various sugars and cells harvested for β-gal activity assays (Fig. 3B). No β-gal activity could be detected in wild-type cells under any growth condition. Activity of the reporter gene with P_slp_ and P_man_ followed trends similar to the transcription levels of the native genes the promoters were derived from (Fig. 3A). Reporter activity in P_man_ cells was around 20 times higher when grown on mannitol than on glucose, while activity in P_slp_ cells was around 2.5 times higher than the highest activity in strain P_man_, and showed minimal dependence on growth sugar. Unexpectedly, the highest activity obtained by P_fru_ cells was less than 20% of the highest activity for P_man_, although this still represents a nearly 20-fold increase compared with the very low activity on glucose. The repression of *mtlD* transcription by the presence of other sugars was not as dramatic here as in the qPCR results (Fig. 3A and Fig. S2), but average β-gal activity of cells grown on glucose plus mannitol is still less than half that of cells grown on mannitol alone.

Since β-gal should allow growth on lactose, which *T. kivui* cannot natively utilize, the strains were also tested for their ability to grow on lactose. Wild-type and P_fru_ cells did not grow on lactose, while P_slp_ cells reached an OD_600_ of 1 within a few days (Fig. S3). For strain P_man_, growth was dependent on the pre-culture, with cells grown on mannitol and already expressing the β-gal able to grow very slowly (reaching an OD_600_ of 0.5 after around 1 week), while cells transferred from glucose exhibited no growth.

To determine the threshold inducer concentration (the minimum concentration where increased expression is evident) for P_fru_, cells were grown in glucose to a target OD of approximately 0.5, then induced with 0.1, 0.5, or 20 mM fructose for 2 h, before harvesting cells to measure β-gal activity. A slight increase in activity is evident with 0.1 mM fructose, and a significant increase (*P* < 0.05) with 0.5 mM (Fig. S4), indicating that the threshold for induction is below 0.5 mM. After 2 h, no residual fructose was detectable in the 0.1 and 0.5 mM samples, meaning the inducer had already been consumed. Comparable data for P_man_ are not available because P_man_ is repressed by the presence of other sugars, so pre-culturing cells in the non-induced state was not possible.

### Investigating operon arrangement

**Fig 4:**
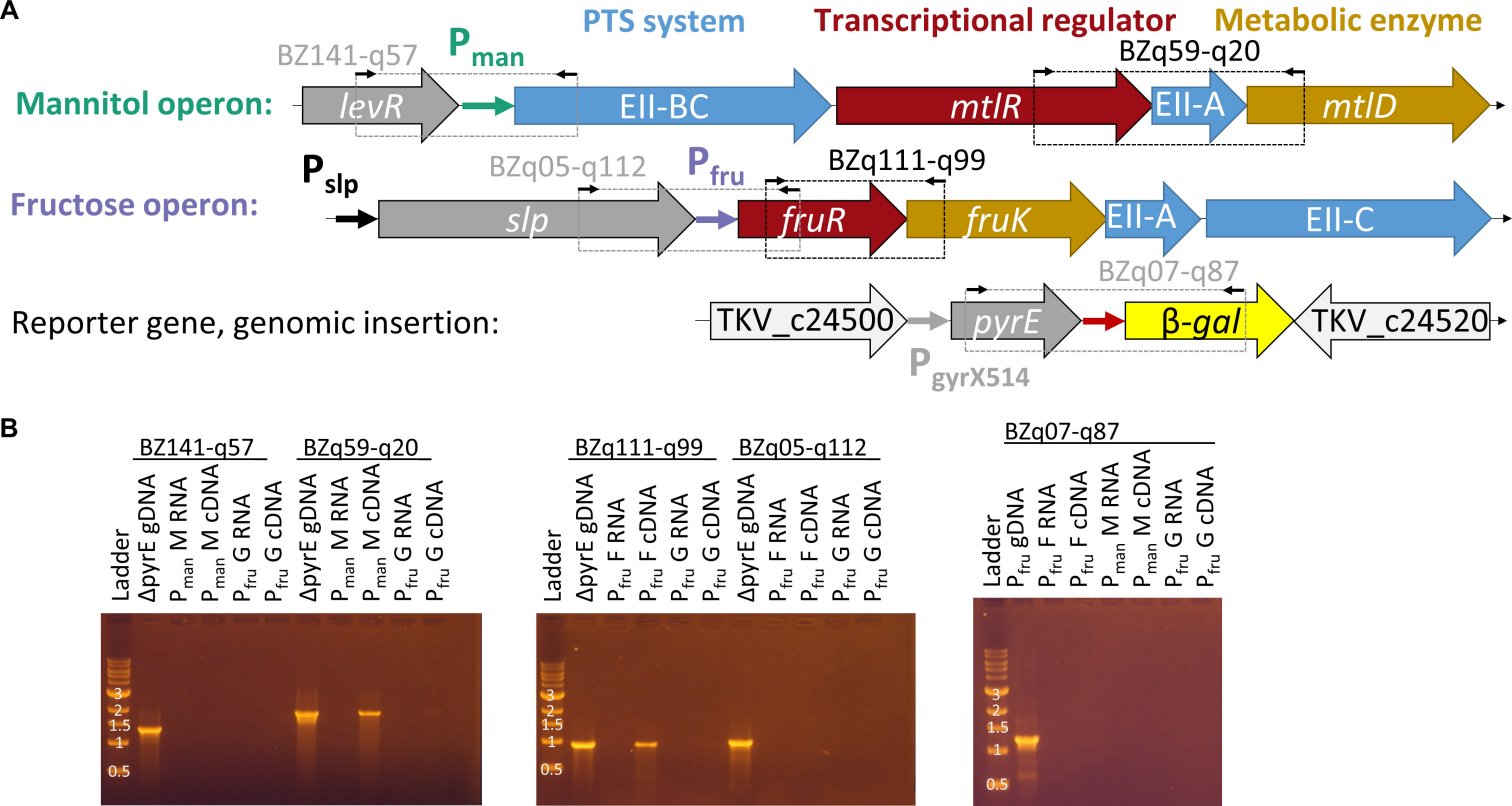
Operon arrangement and co-transcription tests. (**A**) Arrangement of mannitol and fructose operons in the genome of *T. kivui*, as well as the site of genomic insertion of strains with the reporter gene. Colors of large arrows indicate gene function with gene name or product inside the arrow, with red = regulator, orange = metabolic enzyme, and blue genes encoding portions of enzyme II of the PTS system. Medium arrows are promoter regions used in this work, and small black arrows indicate primer binding sites, with boxes indicating the PCR product of two primers. (**B**) DNA agarose gels of co-transcription tests. Each lane is labeled with the strain (P_fru_ or P_man_) from which RNA or DNA was extracted, with RNA and cDNA samples also specifying the sugar cells were grown on (M, G, or F, for mannitol, glucose, or fructose). A band in the cDNA lane indicates co-transcription, while gDNA serves as positive control and RNA (no reverse-transcription) as negative control. The size marker is NEB 1 kb DNA ladder, numbers over bands indicate the length in kilobases.

Interestingly, the gene encoding the S-layer protein *slp* is directly upstream of the fructose operon. Therefore, the native control of the genes involved in fructose metabolism may be influenced by expression of *slp*. To confirm the organization of the fructose and mannitol uptake operons, cDNA generated from cells grown on glucose, fructose, or mannitol was amplified with the primers depicted in Fig. 4A, with genomic DNA as a positive control and RNA as negative control. The results confirm that *slp* and *fruR* are expressed as separate transcripts, while *fruR* and *fruK* formed an operon as expected (Fig. 4B). The P_man_ promoter is preceded by a *levR* gene annotated as a transcriptional regulator, but here again, there was no evidence that this gene was co-transcribed with *mtlR* and *mtlD*. The recombinant strains also contain the selective marker *pyrE* upstream of the reporter gene; however, there was no evidence that the selective marker and reporter gene were co-transcribed. To avoid any possibility of read-through from the *pyrE* gene, its direction was reversed in cloning constructs used for the knockdown constructs described in the next section.

### Use of the inducible promoters to “knockdown” expression of an essential gene

**Fig 5:**
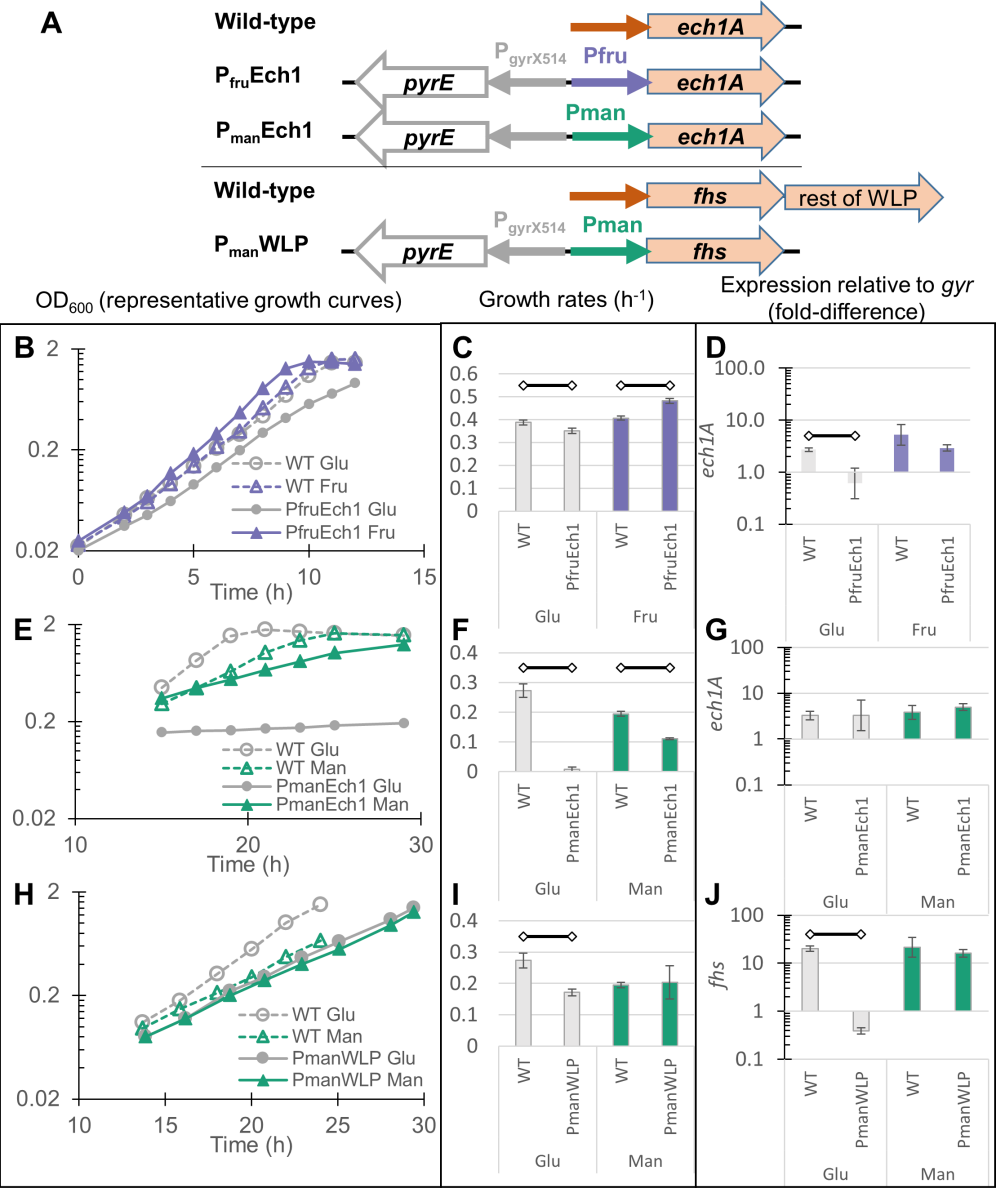
Knockdown of Ech1 or WLP operon reduces growth on non-inducing sugars. Visualization of the promoter regions in *T. kivui* wild type compared with the two knockdown strains, with reversed *pyrE* gene for selection of mutants (**A**). Representative growth curves of P_fru_Ech1 (**B**), P_man_Ech1 (**E**), and P_man_WLP (**H**) on defined medium at 66°C with either glucose (gray circles), fructose (purple triangles), or mannitol (green triangles), all at 30 mM. Because of the much slower growth rates for the P_man_ strains, cells were inoculated to a lower starting OD the night before the experiment and allowed to grow overnight before monitoring began. Open shapes and dashed lines indicate wild-type controls, filled shapes and solid lines are knockdown strains. Average growth rates of P_fru_Ech1 (**C**), P_man_Ech1 (**F**), and P_man_WLP (**I**). Expression levels of the knocked-down gene relative to the *gyrA* reference gene for P_fru_Ech1 (**D**) P_man_Ech1 (**G**), and P_man_WLP (**J**) during growth on glucose (gray), fructose (purple), or mannitol (green). Each bar represents average and standard deviation of at least three biological replicates. Open diamonds connected by black lines indicate comparisons that are significantly different (*P* < 0.05).

A knockout strain of the Ech2 operon has been recently described (18). Genes encoding the Ech1 complex, however, could not be knocked out after multiple attempts, suggesting that the function of Ech1 is essential for *T. kivui*. Therefore, we decided to replace the promoter of the Ech1 operon (450 bp upstream of gene TKV_c01230) with the characterized sugar inducible promoters, generating strains P_fru_Ech1 and P_man_Ech1 (Fig. 5A). The WLP also appears to be essential, since a mutant devoid of the first enzyme of the WLP, the formate-producing hydrogen-dependent carbon dioxide reductase (HDCR), was incapable of growth unless formate was supplemented (36). Most genes of the WLP in *T. kivui* cluster together (37), and the RNAseq data suggest a large gene cluster starting with formyl-THF synthetase (*fhs*, TKV_c19930), catalyzing the next step in the WLP after HDCR, is co-regulated. Therefore, the promoter region (400 bp upstream) of *fhs* was replaced with the mannitol promoter to generate strain P_man_WLP (Fig. 5A).

All three knockdown strains grew slower on glucose than on the inducing sugar (Fig. 5B,C, E, F, H and I), which is especially noteworthy for the P_man_ strains because the wild-type grows faster on glucose than on mannitol (18). All three also grew significantly slower on glucose than the wild type (Fig. 5C, F and I). The growth defect on glucose was especially strong for P_man_Ech1 (Fig. 5F). Despite the slow growth rate, the P_man_Ech1 cells grown on glucose did eventually reach final OD_600_ values above 1.0, but this required incubation for 4 to 5 days.

Quantitative PCR was used to quantify transcription of the knocked-down genes, which confirmed that expression was significantly lower in P_fru_Ech1 and P_man_WLP during growth on glucose (Fig. 5D and J). The results of qPCR for P_man_Ech1 on glucose were noisy (evident from the large error bar in Fig. 5G), which is likely a result of the very slow growth on this condition. To confirm that the previously identified sugar-responsive genes were behaving as expected in the knock-down strains, their expression was likewise quantified with qPCR. The results confirmed that *mtlD* and *fruK* were upregulated in response to mannitol and fructose in the mutants exactly as in the wild type (Fig. S5). The only exception was for P_man_Ech1 on glucose, where *mtlD* expression was again noisy (Fig. S5B), mirroring *ech1A* expression in Fig. 5G.

P_man_WLP showed growth on glucose that was only slightly slower than the wild type, while growth of the two strains on mannitol was essentially identical (Fig. 5I). Expression of *fhs* was reduced 40-fold in the knockdown strain on glucose relative to growth on mannitol, while on mannitol, *fhs* expression in the mutant was as high as in the wild-type (Fig. 5J). It is interesting that the P_man_ promoter was capable of expressing *fhs* at native levels, suggesting the genomic context of *fhs* is important for its high expression.

**Fig 6:**
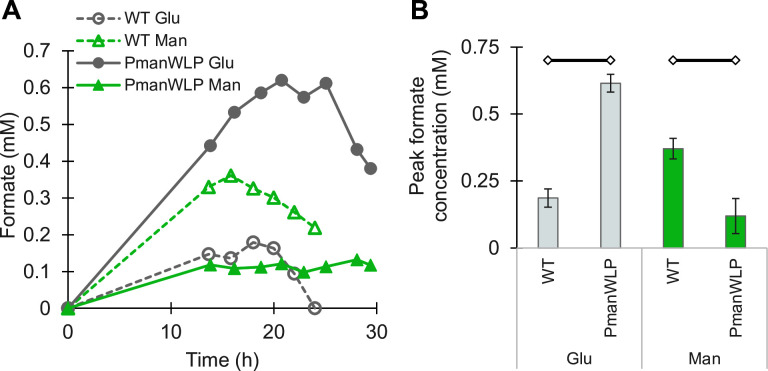
Knockdown of the WLP operon leads to formate accumulation. Representative curves of extracellular formate concentration over time (**A**) and average peak extracellular formate concentration of at least three biological replicates (**B**). Determined by HPLC from cultures of P_man_WLP (solid lines) or wild-type cells (dashed lines) grown on glucose (gray) or mannitol (green) from Fig. 5H. Open diamonds connected by black lines indicate comparisons that are significantly different (*P* < 0.05).

Since the genes of the WLP operon (starting with *fhs*) consume formate generated as an intermediate by HDCR, P_man_WLP was expected to accumulate formate under the knock-down condition (growth on glucose). Low concentrations of formate accumulated in the medium under all growth conditions, peaking in late exponential phase before gradually declining (Fig. 6A). As expected, P_man_WLP accumulated significantly more formate growing on glucose compared with mannitol (Fig. 6B). The wild type produced more formate on mannitol than on glucose, but always less than P_man_WLP in the knockdown condition.

## DISCUSSION

Previous recombinant gene expression in *T. kivui* has always relied on one of a handful of constitutive promoters such as P_slp_, promoter of the S-layer protein (Tkv_c23170), P_gyrX514_ from the gyrase gene of the closely related *Thermoanaerobacter sp*. X514, and P_kan_, the native promoter of the *Staphylococcus aureus* kanamycin resistance gene used in plasmid pMU131. Expression levels with P_slp_ tend to be very high, while P_gyrX514_ and P_kan_ result in weaker expression that is nonetheless adequate to allow growth on selective media when controlling the appropriate marker gene (*kanR* or *pyrE*) (28, 38). More recently, a broader array of promoters was characterized based on their ability to induce expression of a fluorescent reporter gene (27), but this method failed to detect the activity of weaker promoters. Here, we established a recombinant beta-galactosidase reporter gene with higher sensitivity at the T_opt_ (66°C), advantageous for evaluating promoters of intermediate and low strength. It was even capable of quantifying the very low uninduced activity of P_man_ and P_fru_ (Fig. 3B).

The unexpectedly low reporter gene activity of P_fru_ (compared to the promising qPCR data) was potentially due to the selection of the promoter region. For this study, the intergenic sequence upstream of a particular gene was selected as its “promoter region,” but this length varied depending on the gene in question, with P_fru_ the shortest at 170 bp. Promoter function is known to be affected by the surrounding genomic context, both upstream and downstream of transcription initiation, and shorter or “minimal” promoters appear to be particularly sensitive (39). Since promoter strength can also vary with the specific gene being expressed, it is possible that activity with a different reporter gene would be stronger than observed here with *A. bescii* β-galactosidase. The threshold fructose concentration for induction of P_fru_ is certainly below 0.5 mM (Fig. S4) but could not be determined precisely because of the challenge that the inducer is consumed by the cells. Findings with sugar-inducible promoters using non-metabolizable inducers like IPTG (threshold <10 µM [40]) or single cell analysis of very dilute cultures (thresholds ranging from 0.1 to 100 µM [29]) suggests that the true value may be much lower than the upper bound of 0.5 mM that we were able to determine here.

While the general trend of reduced growth rate and gene expression under knockdown conditions was consistent for all three knockdown strains, the extent of the effect varied. Growth of P_fru_Ech1 was only slightly slower on glucose compared to fructose (Fig. 5C), while P_man_Ech1 grew dramatically slower under the knockdown condition (Fig. 5F). P_man_WLP did grow slower than the wild-type on glucose, but there was no significant difference between growth on glucose vs mannitol (Fig. 5I), despite the fact that expression of *fhs* was down-regulated more than 40-fold on glucose according to qPCR (Fig. 5J). The virtually identical growth rate of P_man_WLP on glucose relative to mannitol is interesting, since formate concentrations clearly indicate a bottleneck in the WLP when growing on glucose (Fig. 6). Intracellular formate concentrations in *T. kivui* are not known, but the related thermophilic acetogen *Moorella thermoacetica* has an Fhs with a k_m_ for formate of around 0.5 mM (41). However, resting cells of wild-type *T. kivui* can generate significantly higher concentrations of extracellular formate from H_2_ + CO_2_, especially with excess bicarbonate (42). The combination of these conditions and knockdown of Fhs may result in even higher levels of formate production in the P_man_WLP strain.

The exchange of the *ech1A* promoter demonstrated that Ech1 plays a role in organoheterotrophic growth on glucose. The weak growth of un-induced (glucose-grown) P_man_Ech1 strongly hints that Ech1 expression is limiting growth, although qPCR data were noisy (Fig. 5E), likely because very slow growth disrupted the relationship between expression levels of the reference gene (*gyrA* expression is tied to growth) and the gene of interest. In light of the strong knockdown phenotype in P_man_Ech1, the relatively minor difference in growth rates between induced and non-induced P_fru_Ech1 was surprising, but may reflect general differences in sugar metabolism, since the wild type consistently grows better on fructose than on mannitol. Similar to the growth behavior observed in the ∆Ech2 strain grown on pyruvate (18), P_fru_Ech1 may have accumulated an as-yet unidentified suppressor mutation, or other adaptive mutations, which mitigate the growth defect of the “knockdown” condition.

One obvious limitation of the inducible promoters identified here is that an inducing sugar is required, meaning they cannot be induced under strictly lithoautotrophic conditions. However, as shown here in the knockdown experiments, such promoters do have applications for reducing the undesired activity of essential genes during product formation. In this application, cells would be grown under heterotrophic conditions to accumulate biomass, then switched to H_2_+CO_2_ or syngas for a production phase, where minimization of cell growth would lead to improved product yields. The promoters could also be used for gene induction during growth on a combination of gasses and sugars, since acetogens show promise for the mixotrophic production of chemicals (43). Future work will continue to search for promoters that are more strongly inducible or that can be induced in the absence of organotrophic growth substrates.

In this proof-of-concept study, we established a very sensitive recombinant reporter gene in the thermophilic acetogen *T. kivui* and applied it to characterize two sugar-inducible promoters. We also confirmed they were capable of regulating essential native enzymes. The described promoters have applications in both basic research to better understand acetogenic metabolism, as well as in applied metabolic engineering efforts to utilize *T. kivui* for industrial applications.

## MATERIALS AND METHODS

### Strains, growth experiments, and metabolite analysis

*A. bescii* was cultured in DSMZ 516 medium, supplemented with 20 mM cellobiose from a 0.5 M anoxic stock. *T. kivui* wild type (DSM2030) and ∆*pyrE* (TKV002, [28]) strains were grown several slightly modified variations of DSMZ 171 medium, including defined, complex, and agar-solid mediums (28). A full list of strains is in Table S3. All growth experiments were carried out at 66°C in water baths or incubators.

Growth experiments of knockdown strains were performed in defined medium with 30 mM of either glucose or the inducing sugar, inoculated from cells grown under the same conditions. For the slower-growing strains P_man_WLP and P_man_Ech1, cells were inoculated to a low starting OD_600_ (0.005) and incubated overnight before starting the experiment. Growth was followed by determination of OD_600_, and metabolites were analyzed by HPLC as described (18).

### RNA sequencing

Wild-type *T. kivui* cells were grown in triplicate in complex medium containing either 25 mM glucose or 25 mM mannitol. One milliliter of culture was harvested in mid- to late-exponential phase (OD = 1.1 for glucose, 1.3 for mannitol), rapidly cooled, centrifuged, and the cells kept frozen until RNA extraction.

RNA isolation, library preparation, mapping, and analysis were performed as described (44). Sequencing was performed on the HiSeq4000 instrument (Illumina Inc., San Diego, CA, USA) using HiSeq4000 Reagent Kit sequencing in 50 bp single read mode. Genes with a log2-fold change of +2/−2 and a *P*-adjust value of <0.001 were considered differentially expressed. Raw reads have been deposited in the Sequence Read Archive as SRR28537724–SRR28537729.

### Generation of *T. kivui* mutant strains

All mutants were based on the ∆*pyrE* parent strain TKV_MB002 as described (28). Cloning fragments were amplified with Q5 high-fidelity polymerase (New England Biolabs, Ipswich, MA, USA), while OneTaq polymerase (New England Biolabs) was used for screening of plasmids and mutant strains. A full list of strains is in Table S3, sequences of all primers are listed in Table S4.

For reporter gene strains, linear PCR cloning constructs containing a copy of the native *T. kivui pyrE* gene under the control of the *T. sp*. X514 gyrase promoter, followed by a terminator sequence and the reporter gene (Athe_1927) under control of one of the tested promoters, was inserted into a previously identified quiescent region of the genome. For the generation of pTkv141, the reporter gene was amplified from *A. bescii* genomic DNA with primers BZ170 and BZ171, while a plasmid backbone containing the flanking regions, *pyrE* gene, P_slp_ (the 220 bp upstream of *slp*), and an N-terminal His6-tag, was amplified from pJM009 (45) with primers BZ172 and BZ173. For P_fru_ the native promoter of the *T. kivui* fructose uptake operon was amplified with primers BZ176 and BZ177, while the entirety of pTkv141, except for the P_slp_ promoter, was amplified with primers BZ174 and BZ175. For P_man_ the *T. kivui* mannitol promoter was amplified with BZ184 and BZ185, and the pTkv141 backbone was amplified with BZ182 and BZ183. Transformation was performed with 1 µg of linear cloning fragment amplified from the plasmids with primers BZ168-169. The resulting numbered mutant strains are referred to in the text by the promoter used to control the reporter gene (strain TKV_MB141, P_slp_; TKV_MB145, P_man_; and TKV_MB142, P_fru_). All mutant strains retain *pyrE* and are therefore capable of growth on defined media.

The method to generate strains with the native promoters of the Ech1 and Wood-Ljungdahl pathway operons substituted for the inducible P_man_ or P_fru_ was performed as described above, with the following modifications. Flanking regions to replace the native promoter of the gene being targeted (*ech1A* or *fhs*) were inserted, and the direction of the P_gyrX514_-*pyrE* cassette was reversed, so that it pointed away from the inducible promoter. The strains are numbered based on the plasmid used to generate them, but in the text are referred to by the identity of the inducible promoter and the operon it controls (TKV_MB144, P_fru_Ech1; TKV_MB148, P_man_Ech1; and TKV_MB156, P_man_WLP).

### Quantitative RT-PCR

Cells (2 mL) were harvested in mid-exponential phase (OD 0.4–0.7), rapidly cooled on ice, centrifuged at 15,000 × *g* for 5 min, and the pellets stored at −70°C until RNA extraction. RNA was extracted using the QIAGEN (Hilden, Germany) RNeasy kit with on-column DNase-digestion. Lysis was performed in a FastPrep-24 bead beater (MP Biomedicals, Irvine, CA, USA) at 6 m/s for four 40-s cycles, before continuing with the manufacturer’s standard protocol. The on-column DNase-digestion was extended to 30 min to further reduce gDNA contamination.

For the synthesis of cDNA, 10 µL reactions of the Biozym cDNA Synthesis Kit (Biozym Scientific, Hessisch Oldendorf, Germany) containing 100 ng of RNA were incubated at 42°C for 30 min. The no-RT controls consisted of RNA diluted to the same concentration in RNase-free water and incubated in the same way.

Quantitative PCR was performed with a qTower^3^ G cycler (Analytik Jena, Jena, Germany). The Biozym Blue S’Green qPCR Mix was used, with 2 µL of 10 x diluted cDNA or no-RT template in 10 µL reactions. The *T. kivui* gyrase subunit A (*gyrA*, Tkv_c00100) was used as a reference gene, and a no-RT control with the *gyrA* gene was included for each RNA extraction. Sequences of all primers used for qPCR are included in Table S4.

Statistically significant differences in qPCR were determined using a two-way *t*-test of delta-Ct (log2) values, to allow for assumption of equal variance. Ct values were then converted to linear (fold-change) for figures. For *fruK*, *mtlD*, and reporter gene expression, all growth sugars within the same gene or strain were compared, while for knockdown strains, expression was compared with the wild type grown on the same sugar.

### Co-transcription tests

To determine operon structure, RNA extracted for qPCR was further treated with the TURBO DNA-*free* kit (ThermoFisher Scientific, Waltham, MA, USA) to remove traces of residual gDNA. This RNA was then reverse-transcribed, and the resulting cDNA used as template for PCR with primers binding in the upstream and downstream genes. Positive controls consisted of the same PCR reaction with gDNA as template, and negative controls with RNA as template. Primer binding sites are shown in Fig. 4A, and primer sequences are listed in Table S4.

### Enzyme activity assays

A total of 20 mL of cells was harvested at a final OD of 0.8 to 1.2, resuspended in 650 µL of lysis buffer (50 mM Tris-HCl, 100 mM NaCl, pH 7.8) and processed in a FastPrep-24 bead beater at 6 m/s for four 40-s cycles. Lysed cells were centrifuged at 15,000 × *g* for 5 min, and the supernatant (cell extract) was collected.

Protein concentrations were determined by the Bradford method using the ROTI Nanoquant reagent (Carl Roth, Karlsruhe, Germany) in a 96-well plate.

Activity assays were carried out in 1.7 mL tubes containing 600 µL of substrate solution (4.67 mM pNP- β-gal, dissolved in 58 mM sodium acetate buffer, pH 5.8). Tubes containing substrate were pre-heated in a 65°C heat block for 5 min, then 100 µL of diluted cell extract was added, and 100 µL samples were taken at various time points into wells of a 96-well plate containing 100 µL of 1 M Na_2_CO_3_ to stop the reaction. The concentration of pNP was determined using a standard curve consisting of serial dilutions of a reaction that was allowed to go to completion, and change in the pNP concentration over time was used to calculate specific activity of each cell extract (µmol/min/mg).

## Data Availability

The raw data used to generate Fig. 2, 3, 5, and 6 are provided as supplemental material. RNA sequencing fold-change results for all genes are provided in Table S1, and raw reads have been deposited in the Sequence Read Archive as SRR28537724-SRR28537729.

## SUPPLEMENTAL MATERIAL

Data Set S1 aem.01641-25-s0001.xlsx Raw data.

Supplemental material aem.01641-25-s0002.pdf Tables S2 to S4; Fig. S1 to S5.

Table S1 aem.01641-25-s0003.xlsx RNA sequencing results comparing growth of wild-type *T. kivui* in complex medium at 66°C on mannitol vs. glucose (complete results). Differences of log2 fold-change > 2, with *P*-adj ≤ 0.001, were considered significant.

ASM does not own the copyrights to Supplemental Material that may be linked to, or accessed through, an article. The authors have granted ASM a non-exclusive, world-wide license to publish the Supplemental Material files. Please contact the corresponding author directly for reuse.
