# Supplementary material for: Sugar-inducible promoters for manipulation of core metabolic pathways in the thermophilic acetogen Thermoanaerobacter kivui
Source: Appl Environ Microbiol. 2025 Nov 20;91(12):e01641-25. doi: 10.1128/aem.01641-25 (PMC12724272; doi:10.1128/aem.01641-25)

Supplemental data for:

Sugar inducible promoters for manipulation of core metabolic pathways in the thermophilic acetogen *Thermoanaerobacter kivui*

Benjamin Zeldes, Sabina Mittelstedt, Christoph Baum, Adilia Shakirova, Anja Poehlein, Rolf Daniel, Mirko Basen

Accompanying .xlsx file:

Table S1: Complete RNAseq gene expression data

Table S2 Selected RNA sequencing results comparing growth of wild-type *T. kivui* in complex medium at 66°C on mannitol vs. glucose. Genes with log2 fold-change > 2 and Padj ≤ 0.001 were considered differentially regulated.

LocusTag	Annotation	base Mean	log2 Fold Change	padj
**up on mannitol**				
TKV_c01950	ferrous iron transport protein A	4,325	**3.5**	**< 0.000**
TKV_c01960	ferrous iron transport protein B	53,293	**3.6**	**< 0.000**
TKV_c02390	fructose 1,6-bisphosphatase	30,270	**2.8**	**< 0.000**
TKV_c02840	transcriptional regulator MtlR	3,331	**2.6**	**< 0.000**
TKV_c02850	mannitol-specific PTS enzyme IIA component MtlF	641	**2.5**	**< 0.000**
TKV_c02860	mannitol-1-phosphate 5-dehydrogenase MtlD	1,947	**2.2**	**< 0.000**
TKV_c03470	ATP-binding transport protein NatA	471	**2.2**	**< 0.000**
TKV_c10280	cobaltochelatase CobN subunit	169,889	**2.6**	**0.001**
TKV_c10510	hypothetical protein	394	**2.3**	**< 0.000**
TKV_c10530	phosphoenolpyruvate synthase PpsA	24,468	**2.9**	**0.001**
TKV_c10940	xylose repressor	988	**2.3**	**< 0.000**
TKV_c14030	small acid-soluble spore protein alpha/beta type	201	**2.2**	**< 0.000**
TKV_c17640	Na+/melibiose symporter	275	**2.1**	**< 0.000**
TKV_c17650	hypothetical protein	68	**2.3**	**< 0.000**
TKV_c18170	R-phenyllactate dehydratase activator	82	**2.9**	**< 0.000**
TKV_c18810	pyrophosphate-fructose 6-P 1-phosphotransferase Pfp	11,081	**2.3**	**< 0.000**
TKV_c21440	hypothetical protein	32	**2.5**	**< 0.000**
TKV_c22680	cell wall-associated hydrolase	2,977	**2.1**	**< 0.000**
TKV_c24080	Cd resistance transcriptional regulatory protein CadC	847	**2.2**	**< 0.000**
**up on glucose**				
TKV_c03760	hypothetical protein	80	**-2.1**	**< 0.000**
TKV_c05870	Trp operon repressor family	288	**-2.1**	**< 0.000**
TKV_c08610	hypothetical protein	261	**-2.2**	**< 0.000**
TKV_c14840	tryptophan synthase alpha chain	938	**-2.5**	**< 0.000**
TKV_c14860	N-(5'-phosphoribosyl)anthranilate isomerase TrpF	372	**-2.6**	**< 0.000**
TKV_c14870	indole-3-glycerol phosphate synthase TrpC	788	**-2.7**	**< 0.000**
TKV_c14880	anthranilate phosphoribosyltransferase TrpD	1,136	**-2.6**	**< 0.000**
TKV_c14890	bifunctional protein TrpGD	672	**-2.7**	**< 0.000**
TKV_c14900	anthranilate synthase component 1	2,123	**-2.8**	**< 0.000**
TKV_c17500	beta-lactamase domain-containing protein	1,885	**-2.0**	**< 0.000**
TKV_c17530	cobyrinic acid ac-diamide synthase	2,145	**-2.4**	**< 0.000**
TKV_c17540	dinitrogenase Fe-Mo cofactor biosynthesis protein	732	**-2.1**	**< 0.000**
TKV_c17550	hypothetical protein	991	**-2.7**	**< 0.000**
TKV_c22020	desulfoferrodoxin Dfx	13,171	**-2.4**	**< 0.000**
**Selected non-differentially regulated genes**				
TKV_c00100	DNA gyrase subunit A GyrA	11,600	0.0	0.900
TKV_c01230	Ech-type complex subunit Ech1A	65,610	0.0	0.991
TKV_c13970	phosphate acetyltransferase Pta	47,673	-0.5	**< 0.000**
TKV_c16340	glyceraldehyde-3-phosphate dehydrogenase Gap	114,809	-0.4	0.023
TKV_c19750	Ech-type complex subunit Ech2D	2,604	-1.0	**< 0.000**
TKV_c19930	formyl-tetrahydrofolate synthetase Fhs	321,840	-1.2	**< 0.000**
TKV_c23160	DeoR family transcriptional regulator	118	0.6	0.008
TKV_c23170	cell surface protein SLP	1,855,343	-0.3	0.007

Table S3: Strains used or generated in this study

Strain	Genetic modifications	Parent strain	Reference
T. kivui DSM2030	NA	NA	(Leigh, Mayer, and Wolfe 1981)
C. bescii DSM 6725^T	NA	NA	(Yang et al. 2010)
TKV_MB002 (Δ*pyrE*)	Δ*pyrE* (TKV_c14380)	*T. kivui* DSM2030	(Basen et al. 2018)
TKV_MB141 (P_{slp})	→$P_{gyrX514}$*pyrE* →P_{slp}β-*gal* (Athe_1927)*	TKV_MB002	This study
TKV_MB142 (P_{fru})	→$P_{gyrX514}$*pyrE* →P_{fru} β-*gal* (Athe_1927)*	TKV_MB002	This study
TKV_MB145 (P_{man})	→$P_{gyrX514}$*pyrE* →P_{man}β-*gal* (Athe_1927)*	TKV_MB002	This study
TKV_MB144 (P_{fru}Ech1)	$P_{gyrX514}$*pyrE* ⟵ →P_{fru}*ech1A* (TKV_c01230)	TKV_MB002	This study
TKV_MB148 (P_{man}Ech1)	$P_{gyrX514}$*pyrE* ⟵ →P_{man}*ech1A* (TKV_c01230)	TKV_MB002	This study
TKV_MB156 (P_{man}WLP)	$P_{gyrX514}$*pyrE* ⟵ →P_{man}*fhs* (TKV_c19930)	TKV_MB002	This study

* inserted in region between TKV_c24500 and TKV_c24520

Table S4: Primers used in this study

**Primer**	**Binds**	**Product**	**Sequence** (lower case letters = non-binding tail for plasmid assembly)
**Cloning**			
BZ146	*T. kivui* gDNA	Ech1 promoter UFR	ctcggtacccggggatccGACAGAAGAAGAAATATACAGGGTTTCTG
BZ147			ggtataaaggatgtcatCACACTCTCCTCGTACAGCTT
BZ148	*T. kivui* gDNA	Ech1 promoter DFR	cgaggagagtgtgATGACATCCTTTATACCCATAATGTTAG
BZ149			gcatgcctgcaggtcgactctagaCTATCAGCCAGAGCATCAAAGTAG
BZ150	*T. kivui* gDNA	P_{fru}	gagtgtgGGACTCTTTTATTTGCTTTAAAACAGGC
BZ151			gggtataaaggatgtcatATCAGATATACTCATATTCATTCACATCC
BZ152	pTkv134	bb, UFR, and DFR	cctgttttaaagcaaataaaagagtccCACACTCTCCTCGTACAGCTT
BZ153			ggatgtgaatgaatatgagtatatctgatATGACATCCTTTATACCCATAATGTTAG
BZ154	gDNA of *T. kivui* (screening)	*ech1A* promoter region	GTAGAAGCAGTGGATAAAGTCATACTG
BZ155			GACCTATAATAGAGGCGCTTAAATAGATG
BZ168	Reporter gene plasmids	linear cloning fragment	TCTCTTATCACCACCTCCACA
BZ169			GCAATTATGGCAATGCGTGG
BZ170	*C. bescii* gDNA	Amplify reporter gene Athe_1927	catcatcaccacATGGGCAAAATAAAGCTGAAAAAATTTTTGC
BZ171			ggactaagcccccatttTTAATCTTTTAATCTTCTCAGTATCATAACCTCC
BZ172	pJM009	bb, UFR and DFR	gattaaaagattaaAAATGGGGGCTTAGTCCC
BZ173			gctttattttgcccatGTGGTGATGATGGTGATGCATACAG
BZ174	pTkv141	bb + UFR, *pyrE*, and DFR	gaatatgagtatatctgatATGGGCAAAATAAAGCTGAAAAAATTTTTGC
BZ175			gaatattattgtactatttcCATTTCTTTGAGATAATAAAATAAGCCCACCTG
BZ176	*T. kivui* gDNA	P_{fru}	cttattttattatctcaaagaaatgGAAATAGTACAATAATATTCAACTATAAAGAGTCC
BZ177			gctttattttgcccatATCAGATATACTCATATTCATTCACATCCA
BZ178	pTkv143	bb, UFR, DFR, P_{fru}	gagtaaaaataggGGACTCTTTTATTTGCTTTAAAACAGGC
BZ179			caggaaattttaaCACACTCTCCTCGTACAGCTTG
BZ180	pJM009	*pyrE*	gaggagagtgtgTTAAAATTTCCTGCTTCCCG
BZ181			ataaaagagtccCCTATTTTTACTCTTCTTTCTGG
BZ182	pTkv141	bb, UFR, *pyrE*, and DFR	gtaaaacttatgcaaaatactATGGGCAAAATAAAGCTGAAAAAATTTTTGC
BZ183			cttatcggcacctgtatCATTTCTTTGAGATAATAAAATAAGCCCACCTG
BZ184	*T. kivui* gDNA	P_{man}	cttattttattatctcaaagaaatgATACAGGTGCCGATAAGGATAGAATAG
BZ185			gctttattttgcccatAGTATTTTGCATAAGTTTTACCTCCTTTTTG

BZ190	Ech1 promoter plasmids	linear cloning fragment	CAGGGTTTCTGTACACGAAGC
BZ191			CTCTTTCGTGATATCCATTGCTGC
BZ196	pTkv144	bb, UFR, pyrE, DFR	ggtaaaacttatgcaaaatactATGACATCCTTTATACCCATAATGTTAG
BZ197			ccttatcggcacctgtatCCTATTTTTACTCTTCTTTCTGGTTAAAGC
BZ198	*T. kivui* gDNA	P_{man}	gaagagtaaaaataggATA CAGGTGCCGATAAGGATAGAATAG
BZ199			ggtataaaggatgtcatAGTATTTTGCATAAGTTTTACCTCCTTTTTG
BZ215	*T. kivui* gDNA	*fhs* UFR, DFR and Promoter	ctcggtacccggggatccGTTGTAACTTCTATGCCATGGGC
BZ216			tgcctgcaggtcgactctagaGGTTATCACATAATCAGCAAGCTTC
BZ218	intermediate to pTkv156	bb, UFR and DFR	gtaaagccgggaagcaggaaattttaaGGTGGGCACACTACAAAAAACG
BZ239			gcaaaaaggaggtaaaacttatgcaaaatactATGGCATTTAAGAGCGATATTGAGA
BZ219	pTkv148	*pyrE* and P_{man}	TTAAAATTTCCTGCTTCCCGG
BZ240			AGTATTTTGCATAAGTTTTACCTCCTTTTTG
BZ221	pTkv156	linear cloning fragment	GGCATTTGTAGTTAAGATTGCTAATGC
BZ222			GGTTGCTATCACGCTGTTACAAC
BZ223	gDNA of *T. kivui* (screening)	*fhs* promoter region	CAGGTGATGTTGTAACTTCTATGCC
BZq28			CTCTTTTGCAAGCTCAATTCCAC
LH001	pUC19 derived plasmids	bb	GGATCCCCGGGTACCGAG
LH006			TCTAGAGTCGACCTGCAG
LH28	gDNA of *T. kivui* (screening)	Insert region Tkv_c24500-520	CAGGCTGTGATAATTTGAGAA
LH29			GGTCACGATTTAAAGGACTTA
**RT-qPCR**			
**Primer**	**Gene name**	**Gene Orf**	**Sequence**
gyr-Fwd	*gyrA*	TKV_c00100	CCAGTTGTGCTTCCTTCTCGATTTCC
gyr-Rev			GCGACAATGCCATCTATGACTTCTCC
BZq05	*slp*	TKV_c23170	GACATACAGAGGGCAAATGATCAAC
BZq06			GTTAAGAAGCACTGTGTTGTCTGTG
BZq19	*mtlD*	TKV_c02860	CTGTGGACAGGATAGTACCGAATGTAG
BZq20			CAAGGTCAGCGACAAGTTCCAC
BZq21	*fruK*	TKV_c23150	GACATAATTAGAGAAATAAAAAGCGGCG
BZq22			CTACTGCATCTTCTAACTCCTGC
BZq28	*fhs*	TKV_c19930	CATTAAATAGATTCCCGACAGACACAG
BZq29			CTCTTTTGCAAGCTCAATTCCAC
Ech1A f	*ech1A*	TKV_c01230	CCTCTTTGCCGGTGTAATGAGTAAGG
Ech1A r			AAGCATGGTAAACGCACCCAAC
**Co-transcription tests** (for exact binding locations see Fig. 5)			
**Primer**	**Gene name**	**Gene Orf**	**Sequence**
BZ141	*mtlA*	TKV_c02830	CTTTTGGTGCCATAGCTATCTCTG
BZq07	*pyrE*	(before βgal)	TGACATCAGGAAAACACAGCG
BZq57	*levR*	TKV_c02820	TACACTTTTGCTTGGGGAGACG
BZq59	*mtlR*	TKV_c02840	GCGGTAAATAGAGCTTGCAAGT
BZq99	*fruK*	TKV_c23150	GCATTAGGTCCTATAAAGCCGAGA
BZq111	*fruR*	TKV_c23160	GGAAAGGCGCTTAAAGATTGCA
BZq112	*fruR*	TKV_c23160	AACATTCAATATCGCACCACCG

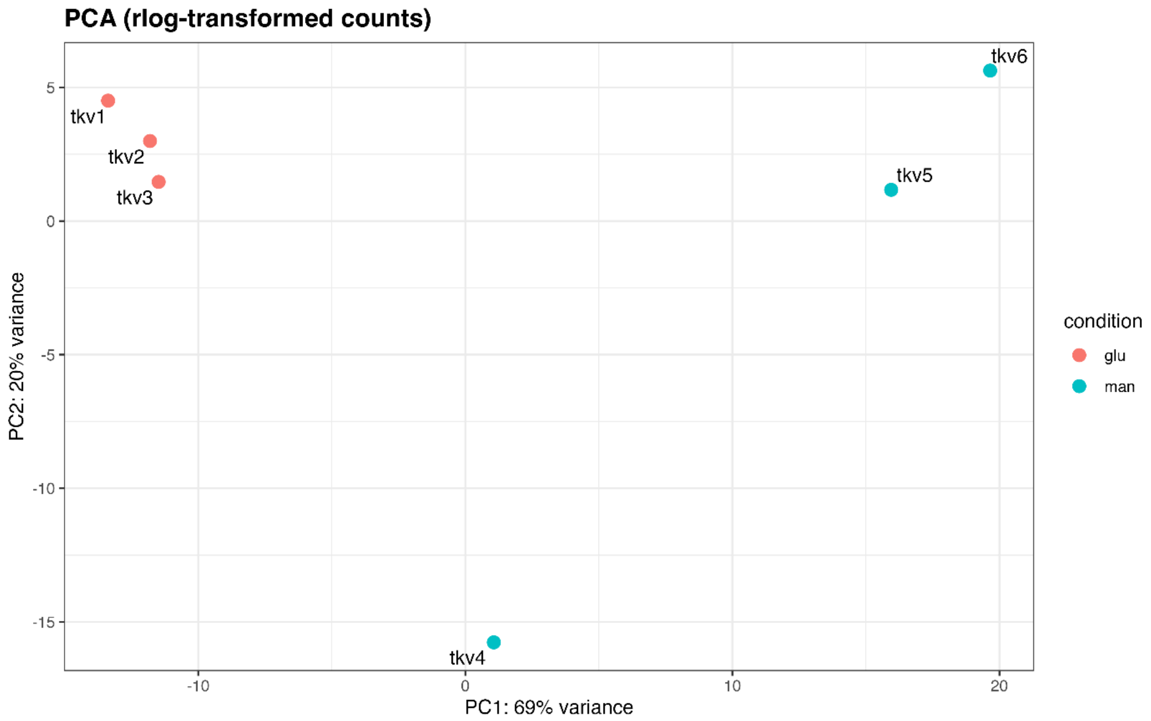

Figure S1: Principle component analysis of RNAseq samples. The three glucose samples (tkv1-3) are shown in red and mannitol samples (tkv4-6) in blue. Glucose and mannitol growth are well separated on PC1. One mannitol replicate is an outlier on PC2, but expression of mannitol-specific genes is consistent across all three replicates (**see Table S1**).

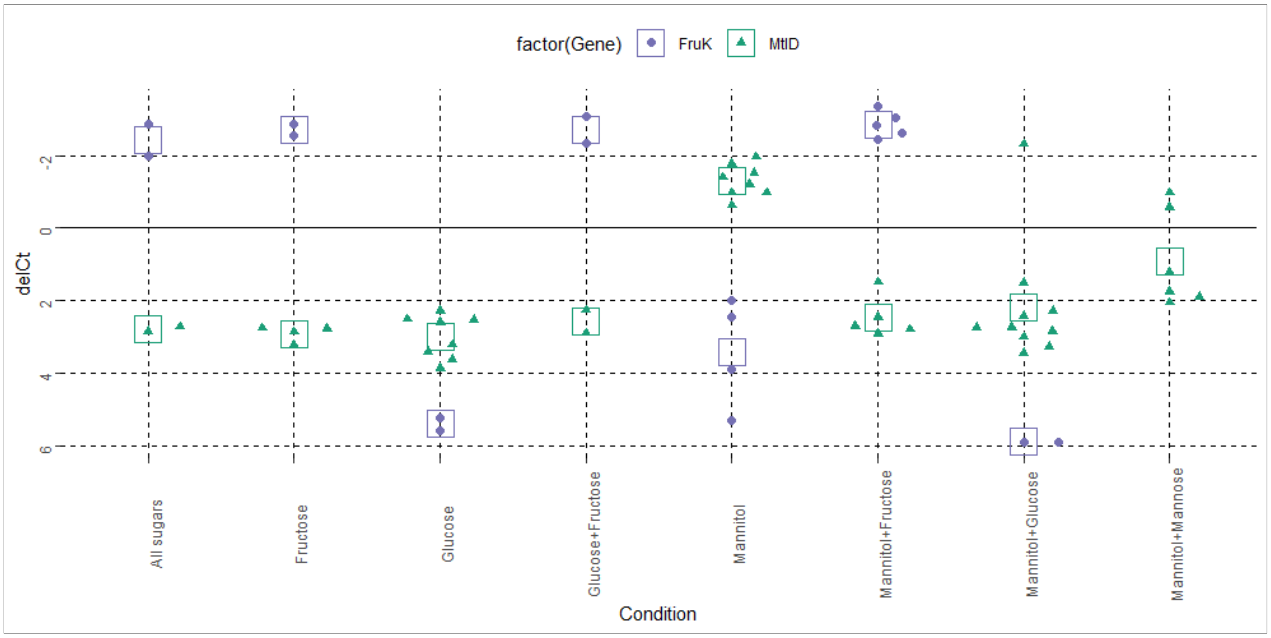

Figure S2: Expression levels relative to *gyrA* reference gene (ΔCt), so negative numbers indicate higher expression (gene reached Ct earlier than the reference gene). Each *n**Ct difference = 2^*n* fold-difference, as DNA concentration doubles every 1 PCR cycle. Each individual data point (*fruK* = green triangles, *mtlD* = purple circles) is a single biological replicate, open boxes show the average for each condition. Technical replicates were not performed, since previous experience found minimal variance compared to the differences between biological samples.

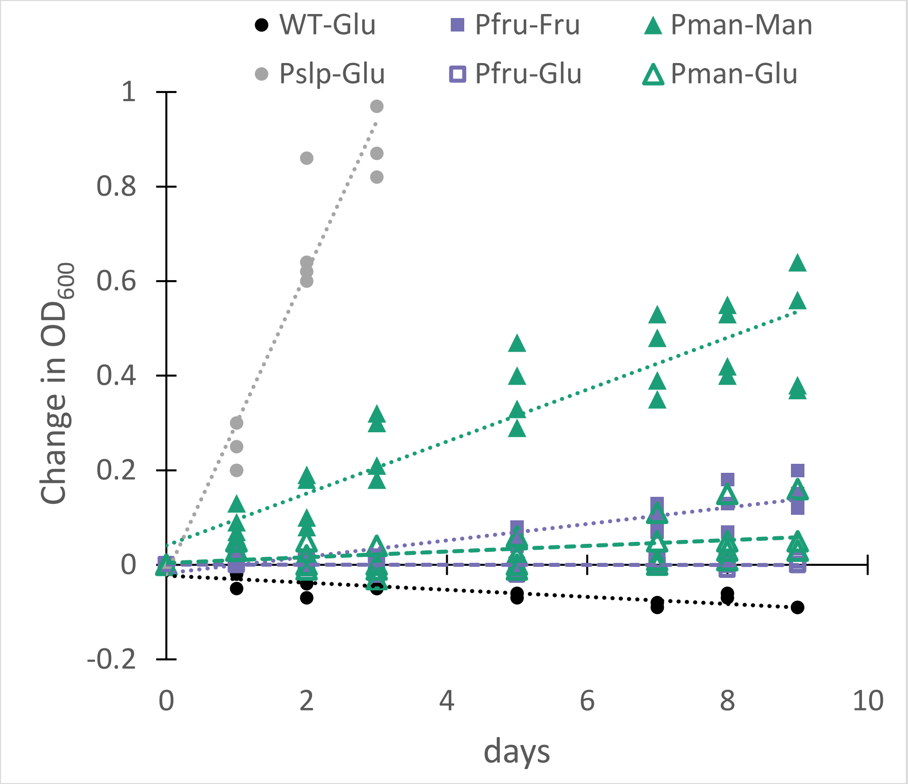

Figure S3: Growth of wild-type and β-galactosidase expressing strains on lactose in defined medium. For P_{fru} and P_{man}, open shapes indicate pre-culture was grown on glucose, while filled shapes were pre-cultured on the respective inducing sugar (P_{slp} was pre-cultured only on glucose). Wild-type = black circles, P_{slp} = gray circles, P_{fru} = purple squares, P_{man} = green triangles.

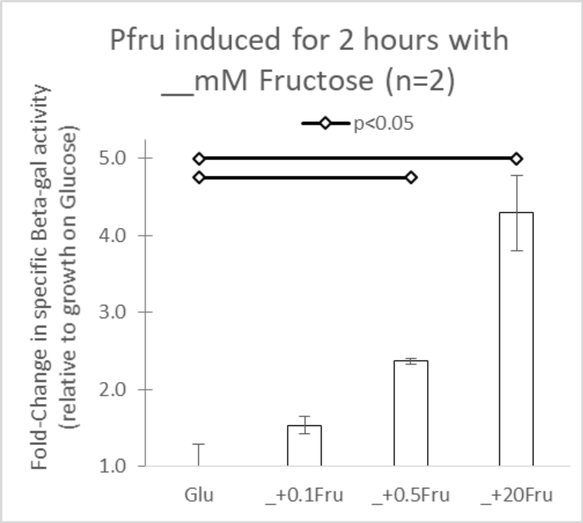

Figure S4: Inducer threshold concentration in P_{fru} cells. Strain P_{fru} was cultured on glucose to a target OD_{600} of ~ 0.5, then fructose (0.1, 0.5, or 20 mM) was added, and cells were cultured for 2 hours before harvesting for β-galactosidase activity assays._ Significantly increased activity is evident in the 0.5 mM fructose samples, indicating that the threshold for P_{fru} induction with fructose is below this value.

Figure S5: Expression of sugar responsive genes in knockdown strains, displayed relative to *gyrA* reference gene (fold-difference). Expression of *mtlD* or *fruK* in wild-type cells grown on either mannitol or fructose compared to glucose and to expression in strains P_{fru}Ech1 (A), P_{man}Ech1 (B), and (C) P_{man}WLP. The same RNA used for qPCR in Fig. 5 was used for this analysis. The lack of significant differences between wild-type and knockdowns on the same sugar confirms that the expected sugar-specific transcriptional response is present in both strains. The one exception is P_{man}Ech1 grown on glucose (B), where the black line indicates $p<0.05$. This can probably be explained by the weak growth under this condition, which led to noisy qPCR results and a very large error bar.

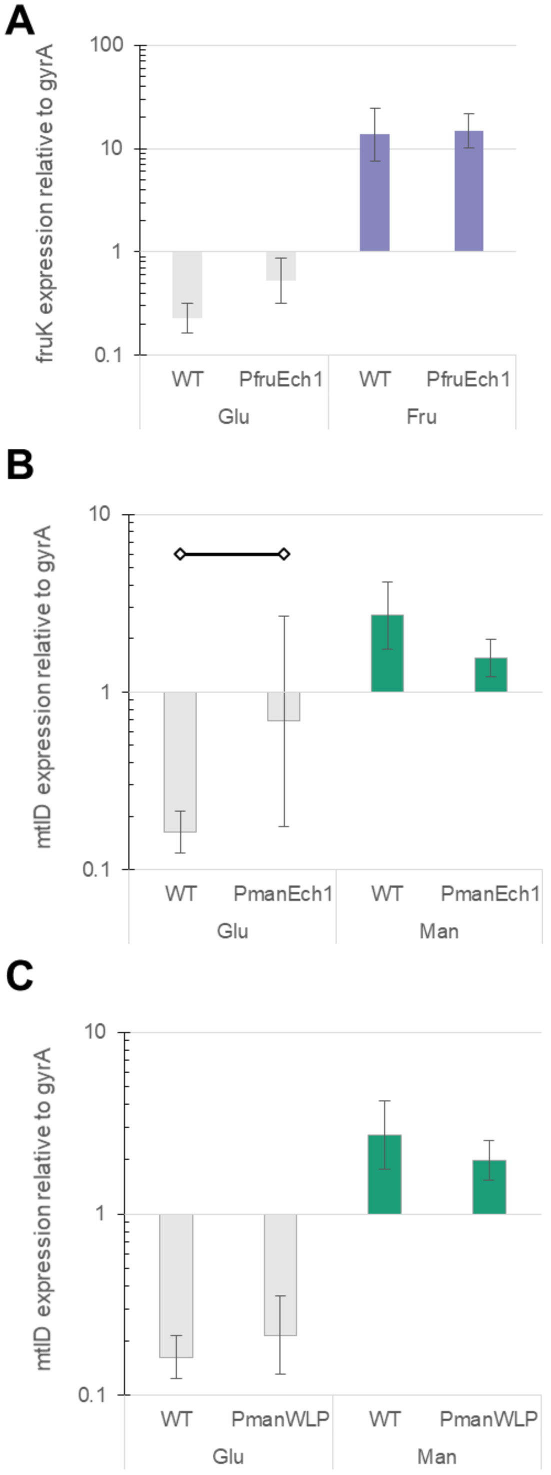

Supplement: Supplemental material — Tables S2 to S4; Fig. S1 to S5. [file aem.01641-25-s0002.pdf]
